# Dyslipidemia in primary care – prevalence, recognition, treatment and control: data from the German Metabolic and Cardiovascular Risk Project (GEMCAS)

**DOI:** 10.1186/1475-2840-7-31

**Published:** 2008-10-15

**Authors:** Elisabeth Steinhagen-Thiessen, Peter Bramlage, Christian Lösch, Hans Hauner, Heribert Schunkert, Anja Vogt, Jürgen Wasem, Karl-Heinz Jöckel, Susanne Moebus

**Affiliations:** 1 Charité – Universitätsmedizin Berlin, Germany; 2 Institute for Clinical Pharmacology, Technical University of Dresden, Germany; 3 Institute for Medical Informatics, Biometry and Epidemiology, University Hospital, University of Duisburg-Essen, Germany; 4 Else Kröner-Fresenius-Zentrum für Ernährungsmedizin, Technical University Munich, Germany; 5 Clinic for Internal Medicine II, University of Lübeck, Germany; 6 University of Duisburg-Essen, Germany

## Abstract

**Background:**

Current guidelines from the European Society of Cardiology (ESC) define low thresholds for the diagnosis of dyslipidemia using total cholesterol (TC) and LDL-cholesterol (LDL-C) to guide treatment. Although being mainly a prevention tool, its thresholds are difficult to meet in clinical practice, especially primary care.

**Methods:**

In a nationwide study with 1,511 primary care physicians and 35,869 patients we determined the prevalence of dyslipidemia, its recognition, treatment, and control rates. Diagnosis of dyslipidemia was based on TC and LDL-C. Basic descriptive statistics and prevalence rate ratios, as well as 95% confidence intervals were calculated.

**Results:**

Dyslipidemia was highly frequent in primary care (76% overall). 48.6% of male and 39.9% of female patients with dyslipidemia was diagnosed by the physicians. Life style intervention did however control dyslipidemia in about 10% of patients only. A higher proportion (34.1% of male and 26.7% female) was controlled when receiving pharmacotherapy. The chance to be diagnosed and subsequently controlled using pharmacotherapy was higher in male (PRR 1.15; 95%CI 1.12–1.17), in patients with concomitant cardiovascular risk factors, in patients with hypertension (PRR 1.20; 95%CI 1.05–1.37) and cardiovascular disease (PRR 1.46; 95%CI 1.29–1.64), previous myocardial infarction (PRR 1.32; 95%CI 1.19–1.47), and if patients knew to be hypertensive (PRR 1.18; 95%CI 1.04–1.34) or knew about their prior myocardial infarction (PRR 1.17; 95%CI 1.23–1.53).

**Conclusion:**

Thresholds of the ESC seem to be difficult to meet. A simple call for more aggressive treatment or higher patient compliance is apparently not enough to enhance the proportion of controlled patients. A shift towards a multifactorial treatment considering lifestyle interventions and pharmacotherapy to reduce weight and lipids may be the only way in a population where just to be normal is certainly not ideal.

## Background

Dyslipidemia is one of the top 5 major risk factors leading to cardiovascular disease. Its treatment has been shown to improve prognosis – morbidity and mortality is substantially reduced in successfully treated as compared to non-treated dyslipidemic controls [1-3].

Although there are differences in defining dyslipidemia and the goals to achieve when treating dyslipidemia there is a general trend to recommend low treatment targets for total cholesterol (TC) and LDL-cholesterol (LDL-C) in all major guidelines (Table 1).

**Table 1:** treatment targets for total cholesterol (TC) and LDL-cholesterol (LDL-C) in the ESC and NCEP guidelines

	Total cholesterol (TC)		Low density cholesterol (LDL-C)	
	mg/dl	mmol/l	mg/dl	mmol/l
**ESC guideline **[4]				
General population	190	5.0	115	3.0
CAD, CVD or DM	175	4.5	100	2.6
**NCEP guideline **[6,5]				
0 or 1 RF			160	4.1
> 2 RF or			130	3.4
CAD event risk < 20%				
CAD or risk equivalent*			100	2.6
optional in very high risk			70	1.8

ESC, European Society of Cardiology; CAD, coronary artery disease; CVD, cardiovascular disease; DM, diabetes mellitus; NCEP, National Cholesterol Education Program; *other clinical forms of atherosclerotic disease, diabetes mellitus, or a 10 year-risk for CAD greater than 20%.

The European guideline on cardiovascular disease prevention in clinical practice for example recommends a TC of below 190 mg/dl (5.0 mmol/l) and an LDL-C of below 115 mg/dl (3.0 mmol/l) for the general population. When additional comorbidity is present (coronary artery disease (CAD), other cardiovascular disease (CVD) or diabetes mellitus) the goals are even lower: < 175 mg/dl (4.5 mmol/l) for TC and < 100 mg/dl (2.6 mmol/l) for LDL-C [4].

The National Cholesterol Education Program (NCEP) guidelines chose another approach to define LDL-C targets based on the presence of additional risk factors: For patients with maximum 1 risk factor LDL-C levels of < 160 mg/dl (4.1 mmol/l) are targeted. Patients with 2 or more risk factors or a 10-year risk for CAD (myocardial infarction or CAD death) of less than 20% LDL-C levels < 130 mg/dl (3.4 mmol/l) are targeted. If patients already show CAD or CAD risk equivalent (other clinical forms of atherosclerotic disease, diabetes mellitus, or a 10 year-risk for CAD greater than 20%) an LDL-C goal of < 100 mg/dl (2.6 mmol/l) is recommended [5] which is identical to the European guideline [4]. The NCEP coordination committee, encouraged by the results of major statin trials, even recommended a goal for LDL-C of less than 70 mg/dl (1.8 mmol/l) in patients at very high risk, at least as a therapeutic option [6].

Available treatments include life-style interventions and pharmacotherapy and these treatments have been shown to successfully alleviate the extent of dyslipidemia if applied rigorously. Statins are highly effective and are prescribed to > 85% of medically treated dyslipidemic patients in primary care in Germany [7,8] either alone (92.9%) or in combination with ezetimibe (3.1%) or fibrates (1.5%). But still there is a substantial gap between the number of patients at risk and the number of patients (successfully) treated to target [7,8] calling for a more in depth understanding of the treatment strategies and pathways, the specific patient type commonly difficult to control and the barriers to overcome in primary care[9]. This could also lead to an improvement in the design of clinical studies to be applicable to a broader population and in particular to the situation in primary care where multiple partly unrelated morbidities meet and treatment is always a trade off between what the guidelines tell and what practice (and budget) allows.

Therefore the dataset of a recent nationwide study with 1,511 physicians (GErman Metabolic and Cardiovascular riSk Project, GEMCAS) documenting 35,869 patients in primary care was used to describe – from a physician perspective -current treatment of dyslipidemia. The following questions were investigated: 1) what is the prevalence of dyslipidemia (treated and untreated) in primary care, 2) what factors are associated with not being recognized, being treated (with or without pharmacotherapy), and finally being controlled if dyslipidemia is known, 3) are there differences with respect to patients in primary prevention compared to high risk patients (high risk patients or pre-existing cardiovascular disease).

## Methods

### Study design and participating physicians

We analysed the dataset of a nationwide cross-sectional prevalence study in primary care. General practitioners and internists with focus on primary health care (GPs) were selected by a stratified, randomized sampling method to receive a random distribution across all German regions. The methods of this study have been described in detail earlier [10].

### Study population

The study population comprised all male and female patients ≥ 18 years of age who visited their GP at the participating sites on the day of the survey regardless of the reason for visiting and who gave their written informed consent to participate. The only reasons for exclusion were conditions that made it impossible for the patient to participate (serious disabilities or diseases, acute emergencies, or pregnancies and breast-feeding within the previous 3 months). The study protocol did not allow for further selection of patients. All patients who were eligible on the given day were included consecutively. In total, 1,511 general practices from 397 out of 438 German cities and administrative districts enrolled 35,869 patients (age range: 18 – 99, women 61.1%).

### Laboratory analysis

An initial screening blood glucose quick test from a capillary (finger stick) was performed in every patient and the results were documented in the reporting form. Additionally, for each patient, venous blood samples were collected and shipped within 24 hours to the central laboratory (Labor 28, Berlin, Germany) by an assigned courier service. The blood samples were analyzed for levels of glucose, LDL-cholesterol, HDL-cholesterol, total cholesterol and triglycerides. GPs were equipped from the central laboratory with pre-labelled serum- and natriumfluoride-(NaF) tubes for taking the samples.

### Diagnostic conventions

Dyslipidemia was defined on the basis of the European guidelines on cardiovascular disease prevention in clinical practice [4] using TC and LDL-C to guide treatment. In general TC < 190 mg/dl and LDL-C < 115 mg/dl were regarded as normal (non dyslipidemic). In patients with established cardiovascular disease (items: myocardial infarction/acute coronary syndrome, stroke/TIA, PAD if questionnaire indicated to be present) or type-2-diabetes mellitus thresholds of < 175 for TC and < 100 mg/dl for LDL-C were applied. So if patients exceeded either one of their thresholds, dyslipidemia was assumed. HDL-C and triglycerides were not used to define dyslipidemia. Ongoing treatment or doctors' diagnosis (lipid disorder indicated to be present) was likewise defined as dyslipidemia. Lipid lowering agents were assumed to be necessary in dyslipidemic patients if the absolute risk based on the SCORE Scoring system exceeded 5% [11] or 20% on the PROCAM Score [12].

### Statistical analyses

For the main variables of the study, basic descriptive statistics (number of observations, mean, median, proportions) and corresponding measures of variability (standard deviation, standard error of the mean, 95% confidence intervals) were calculated. Crude and adjusted prevalence rate ratios PRRs and 95% confidence intervals were computed with the SAS procedure GENMOD. All statistical analyses were conducted using the statistical software package SAS 9.1 (SAS Institute, Cary, NC, USA) [13].

## Results

### Sample characteristics

Basis of the present analysis was a sample of 35,869 patients from the GEMCAS study. Patients had a mean age of 51.7 ± 16.1 years and a mean BMI of 26.9 ± 5.2 kg/m^2^. Patients with dyslipidemia were more frequently male, past smokers, had a higher BMI and waist circumference, and had more frequently cardiovascular disease (Table 2). Overall 76.4% of patients in GEMCAS met the criteria of the ESC for the diagnosis of dyslipidemia [4].

**Table 2:** Baseline characteristics of the GEMCAS sample according to the presence or absence of dyslipidemia

	**Total sample^4^**		**No dyslipidemia**			**Dyslipidemia**		
	**N**	**%**	**N**	**%**	**95%CI**	**N**	**%**	**95%CI**
**Gender**	35 551		8 393			27 158		
female	21 711	61.1	5 421	64.6	63.6–65.6	16 290	60.0	59.4–60.6
male	13 840	38.9	2 972	35.4	34.4–36.4	10 868	40.0	39.4–40.6
**Smoker**	34 508		8 184			26 324		
present	8 629	25.0	2 536	31.0	30.0–32.0	6 093	23.1	22.6–23.7
past	10 058	29.1	2 016	24.6	23.7–25.6	8 042	30.6	30.0–31.1
**BMI**	35 469		8 374			27 095		
overweight (BMI 25–29.9)	12 941	36.5	2 233	26.7	25.7–27.6	10 708	39.5	38.9–40.1
obese (BMI 30-)	8 468	23.9	1 289	15.4	14.6–16.2	7 179	26.5	26.0–27.0
**Waist circumference**	35 360		8 344			27 016		
normal^1^	13 327	37.7	4 758	57.0	56.0–58.1	8 569	31.7	31.2–32.3
elevated^2^	8 058	22.8	1 483	17.8	17.0–18.6	6 575	24.3	23.8–24.9
high^3^	13 975	39.5	2 103	25.2	24.3–26.1	11 872	43.9	43.4–44.5
**Cardiovascular Disease^5^**								
overall CVD	5 509	15.5	658	7.8	7.3–8.4	4 851	17.9	17.4–18.3
MI/ACS	2 234	6.6	208	2.6	2.2–3.0	2 026	7.8	7.5–8.1
Stroke/TIA	905	2.7	116	1.4	1.2–1.7	789	3.1	2.9–3.3
PAD	839	2.5	82	1.0	0.8–1.3	757	3.0	2.7–3.2
Heart Failure	1 959	5.7	236	2.9	2.5–3.3	1 723	6.6	6.3–6.9
**Diabetes**								
Type 1 (acc. to GP)	199	0.6	45	0.6	0.4–0.8	154	0.6	0.5–0.7
Type 2 (acc. to GP)	4 274	12.6	492	6.2	5.6–6.7	3782	14.6	14.2–15.1

								

	**mean ± SD**		**mean**		**95%CI**	**mean**		**95%CI**

**Age**	51.7 ± 16.1		42.2		42.6–41.9	54.7		54.5–54.9
**BMI**	26.9 ± 5.2		25.2		25.1–25.3	27.5		27.4–27.6
**Blood Pressure**								
systolic	130.5 ± 19.1		123.7		123.4–124.1	132.6		132.4–132.8
diastolic	80.0 ± 10.6		77.2		77.0–77.4	80.9		80.8–81.0
**Lipids (mg/dL)**								
total Cholesterol	206.8 ± 41.5		162.7		162.3–163.1	220.2		219.8–220.7
HDL-Cholesterol	62.2 ± 17.3		61.3		61.0–61.6	62.5		62.3–62.7
LDL-Cholesterol	128.0 ± 36.6		90.0		89.7–90.4	139.6		139.2–140.0
Triglycerides	152.4 ± 123.5		110.0		108.5–111.5	165.2		163.6–166.8
**Blood sugar (mg/dL)**								
random glucose	98.0 ± 32.2		91.6		91.0–92.1	99.9		99.5–100.3
fasting glucose	96.1 ± 25.5		90.3		89.6–91.1	97.5		97.1–98.0

^1 ^normal WC: male ≤ 94, female ≤ 80 cm; ^2 ^elevated WC: male > 94 & ≤ 102 cm, female > 80 & ≤ 88 cm; ^3 ^high WC: male > 102 cm, female > 88 cm; ^4 ^N = 35869 total patients in study; ^5 ^overall CVD according to GP and/or patient. Spec. CVD according to GP

### Prevalence of dyslipidemia by age group and gender

**Figure 1:**
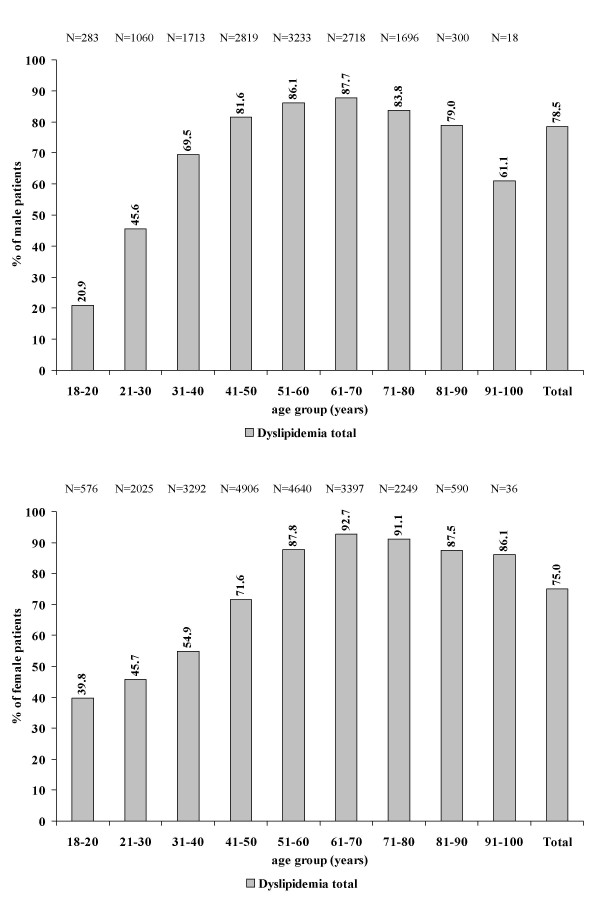
**Proportion of patients in different age groups being dyslipidemic as the the 2003 ESC guidelines**. Total dyslipidemia is defined as either "lipid disorders yes" or "lipid lowering agent yes" or lab values compatible with the diagnosis of dyslipidemia as previously defined. Reference for each percent number given is the total number of patients in that age group indicted in the upper part of the figure.

Dyslipidemia, based on the current ESC guideline definition, was a frequent condition in all age groups from 18 up to 100 years (Figure 1). While the proportion of dyslipidemic patients was low in the young age group (20.9% in male and 39.8% in female patients up to an age of 20 years) it peaked in the age group of 61 – 70 years in both genders with a gradual decline thereafter, more so in male than in female patients.

### Characteristics of patients with diagnosed dyslipidemia

**Figure 2:**
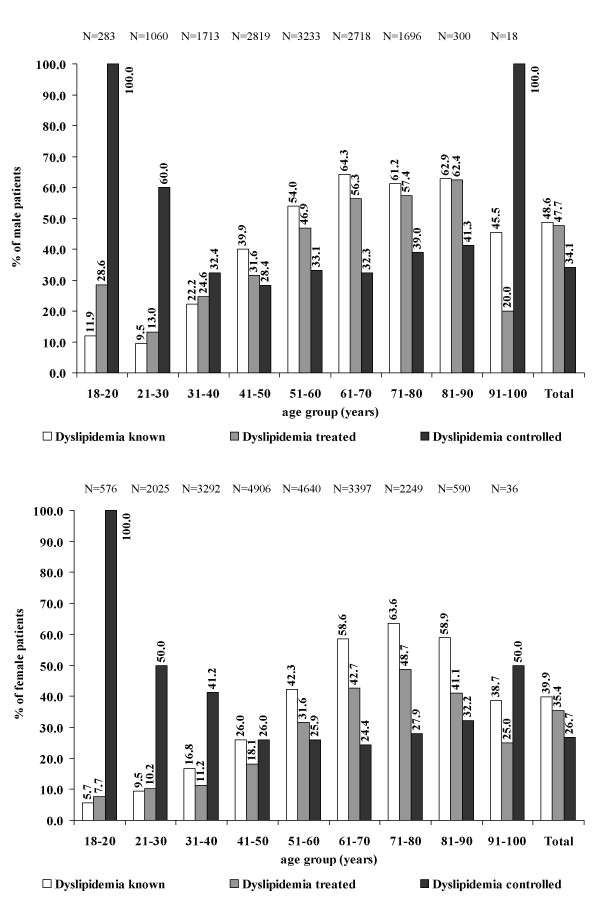
**Known, treated and controlled dyslipidemia in primary care**. Known dyslipidemia is defined as either "lipid disorders yes" or "lipid lowering agent yes". Treated dyslipidemia is defined as "lipid lowering agent yes". Controlled dyslipidemia is defined as "lipid lowering agent yes" and lab values within the limits previously defined. Reference for each percent number given is the number of patients in the previous category, e.g. 11.9% of the 20.9% dyslipidemic patients in the age group 18–20 are known to be dyslipidemic by their treating physician.

The proportion of patients whose dyslipidemia was known to the treating physician was between 9.5 and 64.3% in men, and 5.7 and 63.6% in women. Highest rates were seen in men between 61 and 70 years, the lowest in female patients between 18 and 20 years (Figure 2). Table 3 displays that the proportion of diagnosed patients was generally higher in women than in men (PRR 1.15 [95%CI 1.12–1.17]), likewise this was true for higher age (PRR 1.68 [95%CI 1.64–1.72]), risk factors like type-2-diabetes (PRR 1.51 [95%CI 1.41–1.58]), hypertension (PRR 1.70 [95%CI 1.65–1.76]), and a high BMI (PRR 1.48 [95%CI 1.42–1.53]) and WC (PRR 1.50 [95%CI 1.44–1.56]). Patients with cardiovascular end organ damage (cardiovascular disease, PAD, stroke, myocardial infarction, and heart failure) had more often a diagnosis of dyslipidemia. The same was true for patients with liver disease (PRR 1.57 [95%CI 1.47–1.68]).

**Table 3:** Comparison of patients with a) known vs. unknown dyslipidemia; b) controlled without pharmacotherapy (drugs) vs. uncontrolled and c) controlled with pharmacotherapy (drugs) vs. uncontrolled

	**Known vs. unknown dyslipidemia**			**Controlled without drugs vs. uncontrolled**			**Controlled with drugs vs. uncontrolled**		
	**N**	**PRR**	**95%CI**	**N**	**PRR**	**95%CI**	**N**	**PRR**	**95%CI**
Male gender vs. female	5 296	1.15^1^	1.12;1.17	181	2.00^1^	1.60;2.50	663	1.30^1^	1.67;1.44
Age > 65 years vs. ≤ 65	4 485	1.68^2^	1.64;1.72	110	0.97^2^	0.78;1.22	569	1.14^2^	1.03;1.26
**Risk factors**									
Type-2-Diabetes vs. none	2 840	1.51	1.44;1.58	137	1.37	1.10;1.72	463	0.95	0.86;1.05
Hypertension (140/90) vs. no	3 995	1.70	1.65;1.76	104	1.06	0.83;1.35	337	1.20	1.05;1.37
BMI ≥ 30 vs. < 25 kg/m^2^	3 999	1.48	1.42;1.53	134	1.15	0.87;1.54	361	0.84	0.73;0.97
WC > 102/88 cm vs. normal	6 400	1.50	1.44;1.56	187	0.99	0.76;1.30	588	0.77	0.68;0.87
**Co-morbid disease**									
CVD vs. no	3 609	1.45	1.41;1.50	121	1.34	1.06;1.70	770	1.46	1.29;1.64
MI vs. no	1 695	1.24	1.20;1.29	46	1.48	1.09;2.01	494	1.32	1.19;1.47
Stroke vs. no	607	1.26	1.16;1.37	18	1.08	0.69;1.71	139	1.08	0.93;1.25
PAD vs. no	621	1.33	1.22;1.44	21	1.17	0.76;1.79	147	1.07	0.93;1.24
Heart Failure vs. no	1 284	1.05	1.02;1.08	52	1.78	1.32;2.40	241	1.11	0.98;1.25
Liver disease vs. no	1 436	1.57	1.47;1.68	56	1.29	0.95;1.75	144	0.94	0.79;1.11
**Patient self reported**									
School < 10 years vs. ≥ 10	6 751	1.14	1.11;1.18	181	1.17	0.92;1.48	633	0.89	0.80;0.99
Apprenticeship vs. > Appr.	9 138	1.06	1.02;1.11	244	1.62	1.07;2.47	789	0.80	0.70;0.91
Hypertension vs. no	6 668	1.56	1.51;1.61	180	1.17	0.92;1.49	768	1.18	1.04;1.34
MI vs. no	1 061	1.51	1.41;1.61	36	2.13	1.53;2.96	324	1.37	1.23;1.53
Stroke vs. no	601	1.24	1.14;1.35	17	1.14	0.71;1.82	129	1.07	0.92;1.25
Diabetes vs. no	2 590	1.51	1.45;1.58	119	1.38	1.10;1.74	430	1.01	0.91;1.12
Dyslipidemia vs. no	7 928	3.80	3.64;3.96	145	0.59	0.46;0.75	741	0.70	0.61;0.79
Liver vs. no	933	1.18	1.14;1.21	29	1.10	0.77;1.59	86	1.01	0.84;1.21
Kidney vs. no	968	1.01	0.98;1.03	26	1.14	0.77;1.67	103	1.01	0.86;1.20
Rheuma vs. no	1 235	1.04	1.02;1.06	30	1.15	0.80;1.66	103	0.91	0.76;1.08
Cancer vs. no	917	0.98	0.94;1.02	26	1.23	0.83;1.81	86	1.01	0.84;1.21
Arthrosis vs. no	4 543	1.10	1.07;1.13	97	0.95	0.74;1.22	398	0.87	0.77;0.97
Sports > 4 h vs. ≤ 4 h	941	1.06	1.01;1.12	22	1.05	0.70;1.59	98	0.87	0.74;1.03
Dyspnea on stairs vs. none	5 413	1.12	1.07.1.16	144	1.82	1.05;3.17	544	0.83	0.63;1.10
Smoking ever vs. never	6 222	1.06	1.04;1.09	178	1.16	0.91;1.48	585	0.87	0.78;0.97
Diet many vs. none	343	1.50	1.35;1.68	10	1.39	0.75;2.60	24	0.90	0.64;1.29

PRR, prevalence rate ratios adjusted for age and gender; ^1 ^only age adjusted, ^2^only sex adjusted

Patients' knowledge about cardiovascular risk factors and disease was also associated with a higher proportion of a diagnosis of dyslipidemia (Hypertension, MI, Diabetes, and many dietary attempts).

### Controlled dyslipidemia in patients with life style intervention or no therapy

The overall percentage that dyslipidemia in patients with no pharmacotherapy but life style intervention or no therapy at all is controlled was low (about 10%). The percentage of controlled dyslipidemia increased when patients were male (PRR 2.00; [95%CI 1.60–2.50]), had type-2-diabetes (PRR 1.37 [95%CI 1.10–1.72]), prior MI (PRR 1.48; [95%CI 1.09–2.01]), CVD (PRR 1.34 [95%CI 1.06–1.70]), or heart failure (PRR 1.78 [95%CI 1.32–2.40]). If patients knew about their MI (PRR 2.13 [95%CI 1.53–2.96]), had diabetes (PRR 1.38 [95%CI 1.10–1.74]), or reported dyspnoea on exertion (PRR 1.82 [95%CI 1.05–3.17]) control of dyslipidemia was increased (Table 3).

### Controlled dyslipidemia in patients on pharmacotherapy

Up to 62.4% of male and 48.7% of female patients with known dyslipidemia were treated with pharmacotherapy (Figure 2). There was an age-related increase in both genders until the age group 81 – 90 in men and until the age group 71 – 80 in women. Control rates on pharmacotherapy varied between 28.4 and 100.0% in men and 24.4 – 100.0% in women.

Patients with higher age (> 65 years, PRR 1.14 [95%CI 1.03–1.26]), with hypertension (PRR 1.20 [95%CI 1.05–1.37]), cardiovascular disease like CVD (PRR 1.46 [95%CI 1.29–1.64]), and MI (PRR 1.32 [95%CI 1.19–1.47]) were more likely to be controlled using pharmacotherapy. Patients with a high BMI (PRR 0.84 [95%CI 0.73–0.97]) or WC (PRR 0.77 [95%CI 0.68–0.87]) were less likely well controlled (Table 3). Additionally, patients reporting a low level of education (< 10 years of schooling, PRR 0.89) and apprenticeship only (PRR 0.80) as well as patients reporting to have arthritis (PRR 0.87) or having ever smoked (PRR 0.87) had lowest rates of control.

### Control rates in high risk patients with and without existing CVD

**Figure 3:**
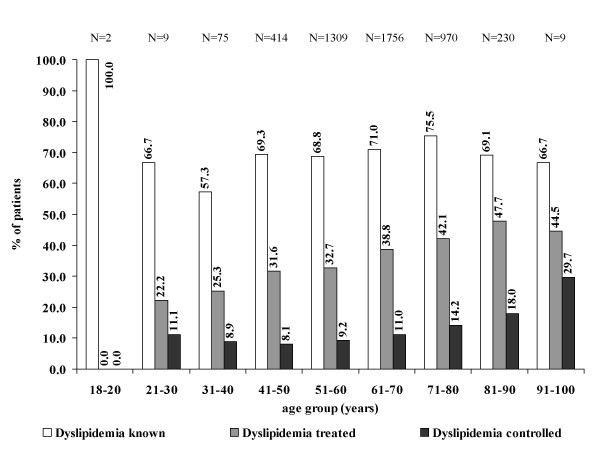
**Dyslipidemia treatment and control in patients with a SCORE Score > 5%, PROCAM Score > 20% or Diabetes mellitus**. Dyslipidemia treatment and control in patients with a SCORE Score > 5%, PROCAM Score > 20% or Diabetes mellitus (equals 100%). Displayed are the proportions of patients with known (left), treated (middle), and controlled dyslipidemia (right column in every age group). Reference for the first category is the total number of patients which are eligible according to the criteria defined; reference for subsequent percent numbers is the number of patients in the previous category.

It is recommended by international guidelines that patients without existing cardiovascular disease (primary prevention) should receive lipid lowering therapy if their SCORE score exceeds 5% or their PROCAM score 20% or when Diabetes mellitus is present [14]. These patients are the basis of Figure 3. Amongst these about 2/3 were known to have dyslipidemia, 1/3 was treated with pharmacotherapy (with an age-dependent increase from 22.2 to 47.7%) and little more than 10% were finally controlled (low 8.1% in the age group 41 – 50, high 18.0 to 29.7% in the elderly).

**Figure 4:**
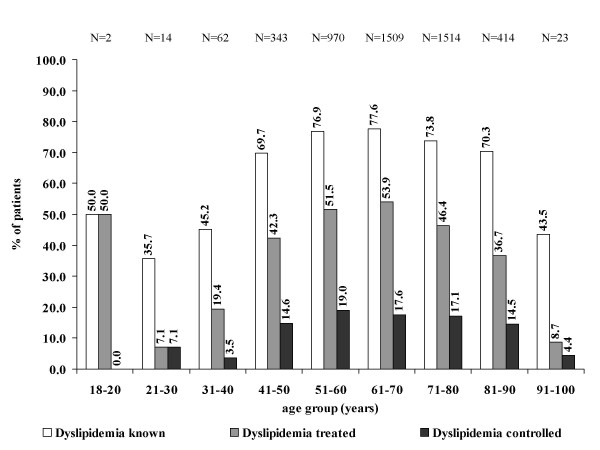
**Dyslipidemia treatment and control in patients with CVD/MI/stroke/PAD and/or heart failure**. Dyslipidemia treatment and control in dyslipidemic patients with CVD/MI/stroke/PAD and/or heart failure. Displayed is the proportion of patients with known (left), treated (middle) and controlled dyslipidemia (right column in every age group). Reference for the first category is the total number of patients which are eligible according to the criteria defined, reference for subsequent percent numbers is the number of patients in the previous category.

Pharmacotherapy is necessary in dyslipidemic patients with pre-existing cardiovascular disease like myocardial infarction, stroke, peripheral arterial disease, or heart failure. These patients are displayed in Figure 4. In these patients, dyslipidemia was usually known (between 50 and 78% per age group), treated up to 50% of cases, and finally controlled in up to about 20%.

## Discussion

Using the ESC 2003 guidance on the treatment of dyslipidemia [4], which was valid at the time of this study, it was documented that dyslipidemia was highly frequent in primary care, in particular in patients beyond the age of 40 years. Dyslipidemia in these patients was almost the rule with prevalence rates of 85%. Many patients with a diagnosis of dyslipidemia were known by their primary care physicians to be dyslipidemic, but despite life style intervention and/or using pharmacotherapy dyslipidemia were not fully controlled to the extent requested by the recent guidelines of the ESC [4]. The chance to be diagnosed and subsequently controlled using pharmacotherapy was generally higher in high risk patients and those with known CVD. This observation is reasonable given the recommendation to start pharmacotherapy in patients in which cardiovascular risk exceeds the threshold of 5% using the SCORE Score (risk of cardiovascular death within the next 10 years) or 20% using the PROCAM Score (risk of cardiovascular disease within 10 years) or in which cardiovascular disease is already present [14].

### Prevalence

In total, we documented a prevalence of dyslipidemia using the ESC criteria of 76.4%. This figure is nominally higher as compared to prevalence rates reported from population-based cohort studies like MONICA [15], PROCAM [16], or GRIPS [17]. Similarly studies from primary care have reported high rates of uncontrolled dyslipidemia [8,9,18,19]. A meaningful comparison with results of other studies is however difficult because of different study populations (patient-based versus population-based), different age-distributions, different and less tight definitions used for dyslipidemia (i.e. TC/HDL-C ratio). Furthermore, there are even differences between the current guidelines in the extent of recommended LDL-C lowering thus giving rise to different prevalence estimates. An example for this is a comparable recent study conducted in primary care (DETECT) which reported prevalence rates for dyslipidemia of approximately 50% based on the NCEP criteria [5,7]. For example, for patients with lower NCEP risk classifications, the European guidelines recommend an optimal LDL-C level of below 115 mg/dl, while the NCEP guidelines recommend an LDL-C below 160 mg/dl. These differences may well account for the different prevalence rates reported.

### Treatment of patients with existing CVD

A number of secondary prevention trials have shown the benefit of lipid lowering in patients with pre-existing cardiovascular disease [20-24]. Furthermore, because of a high baseline risk in these patients the benefit of pharmacotherapy translates into a high absolute risk reduction and in these patients statins are highly cost-effective drugs. This is also the apparent focus in primary care in Germany as patients with cardiovascular risk factors like type-2 diabetes or pre-existing cardiovascular end organ damage are diagnosed, treated and controlled to a higher extent than patients without. Control rates however are not sufficient in patients in which physicians decided to use pharmacotherapy. Only about 55% of male and 40% of female patients are controlled to the extent the ESC guidelines ask for. However, this rate is substantially higher compared to rates in hypertension, where control rates of about 20% have been reported for primary care [25,26].

### Treatment of high risk patients without existing CVD

Primary prevention is warranted only in patients not meeting the currently accepted thresholds of the ESC or NCEP guidelines respectively, unless their risk for cardiovascular death exceeds 5% or the risk of cardiovascular events 20% including patients with diabetes [11,12,14]. This is reasonable given the risk benefit ratio of currently available pharmacotherapy favouring the use of drugs in high risk patients. It is also reasonable because of the price of pharmacotherapy. Considering a substantial proportion of the German population to be dyslipidemic (irrespective whether the ESC or the less tight NCEP guidelines are used as a reference) it would be a substantial cost burden for the health care system with an uncertain outcome. The prices for statins have dropped over the last years, but still the general distribution of statins to every patient appears neither feasible nor wanted.

### Perspectives

It is however unclear whether the currently available pharmacotherapeutic options, despite being highly effective in clinical trials, are sufficient or even suitable in clinical practice to control dyslipidemia in a large proportion of patients. This study clearly shows that reaching prevention targets on a larger scale is very difficult to achieve. Several steps may improve the situation: 1) Physicians could be more aggressive to meet their patients' treatment goals; 2) Patients should be more compliant with therapy; 3) guidelines could be tailored more to the need of physicians in primary care. It may however be questioned whether reaching these lower goals has benefit for the individual or the collective at all. All this points to the fact that dyslipidemia is a multifactorial disease which has to be addressed at multiple levels to achieve long-term control. Prevention aiming at reducing the metabolic syndrome by increasing physical activity and controlling waist circumference has been shown to be highly effective if these steps are installed early enough in the course of a person's life.

## Conclusion

Treatment guidelines of the ESC are difficult to meet in primary care. A gross proportion of patients with an indication for prevention efforts will not meet these treatment recommendations in daily life. A call for more aggressive treatment or higher patient compliance is apparently not enough to enhance the proportion of well treated patients. A shift away from a one-dimensional pharmacotherapy to control dyslipidemia to a multifactorial treatment addressing multiple metabolic issues in these patients considering lifestyle interventions with a reduction of body fat (BMI, WC) and an increase in physical activity may be the only way in a population where just to be normal might not be ideal.

## Competing interests

None of the authors reports a conflict of interest for this manuscript other than that the study has been funded by Sanofi Aventis Deutschland GmbH.

## Authors' contributions

SM planned and performed the study. CL participated in the design of survey instruments and performed the statistical analysis. EST and PB have been writing the manuscript, AV, HS and HH revised the manuscript for important intellectual content; JW participated in the study design. KHJ supervised scientific, ethical and data privacy issues of the study. All authors read and approved the final manuscript.
